# Photodegradation of Aquaculture Antibiotics Using Carbon Dots-TiO_2_ Nanocomposites

**DOI:** 10.3390/toxics9120330

**Published:** 2021-12-02

**Authors:** Vitória L. Louros, Liliana M. Ferreira, Valentina G. Silva, Carla Patrícia Silva, Manuel A. Martins, Marta Otero, Valdemar I. Esteves, Diana L. D. Lima

**Affiliations:** 1 CESAM & Department of Chemistry, University of Aveiro, Campus de Santiago, 3810-193 Aveiro, Portugal; vitorialouros@ua.pt (V.L.L.); mlilianaferreira@ua.pt (L.M.F.); valentinagsilva@ua.pt (V.G.S.); patricia.silva@ua.pt (C.P.S.); valdemar@ua.pt (V.I.E.); 2 CESAM & Department of Environment and Planning, University of Aveiro, Campus de Santiago, 3810-193 Aveiro, Portugal; marta.otero@ua.pt; 3 CICECO & Department of Materials and Ceramic Engineering, University of Aveiro, Campus de Santiago, 3810-193 Aveiro, Portugal; mamartins@ua.pt

**Keywords:** photocatalysts, carbon dots, aquaculture industry, water treatment, solar radiation

## Abstract

In this work, carbon dots (CD) were synthesized and coupled to titanium dioxide (TiO_2_) to improve the photodegradation of antibiotics in aquaculture effluents under solar irradiation. Oxolinic acid (OXA) and sulfadiazine (SDZ), which are widely used in aquaculture, were used as target antibiotics. To prepare nanocomposites of CD containing TiO_2_, two modes were used: in-situ (CD@TiO_2_) and ex-situ (CD/TiO_2_). For CD synthesis, citric acid and glycerol were used, while for TiO_2_ synthesis, titanium butoxide was the precursor. In ultrapure water (UW), CD@TiO_2_ and CD/TiO_2_ showed the largest photocatalytic effect for SDZ and OXA, respectively. Compared with their absence, the presence of CD@TiO_2_ increased the photodegradation of SDZ from 23 to 97% (after 4 h irradiation), whereas CD/TiO_2_ increased the OXA photodegradation from 22 to 59% (after 1 h irradiation). Meanwhile, in synthetic sea salts (SSS, 30‰, simulating marine aquaculture effluents), CD@TiO_2_ allowed for the reduction of SDZ’s half-life time (*t*_1/2_) from 14.5 ± 0.7 h (in absence of photocatalyst) to 0.38 ± 0.04 h. Concerning OXA in SSS, the *t*_1/2_ remained the same either in the absence of a photocatalyst or in the presence of CD/TiO_2_ (3.5 ± 0.3 h and 3.9 ± 0.4 h, respectively). Overall, this study provided novel perspectives on the use of eco-friendly CD-TiO_2_ nanocomposites for the removal of antibiotics from aquaculture effluents using solar radiation.

## 1. Introduction

Aquaculture has become the major source of global food-fish production [1]. In order to satisfy the demand of fish and aquatic species, between 1990 and 2018, global aquaculture production increased by 527% [2]. As in other cultures, organisms produced in every type of aquaculture are prone to disease. Because of that, either for treatment or just as a prophylactic measure, antibiotics are widely employed. Sulfonamides (SAs) and quinolones (QAs) are two families of antibiotics used worldwide for the treatment of a large range of infections [3,4,5,6]. A recent study on the 15 major aquaculture-producing countries revealed that, within SAs, sulfadiazine (SDZ) was the most used antibiotic, with application in 72.7% of these countries [1]. As for QAs, ciprofloxacin (CIP), oxolinic acid (OXA), and enrofloxacin (ENR) led the way, with 45.5% (for both CIP and OXA) and 54.5% (for ENR) application within the 15 considered countries [1]. The overuse of antibiotics has led to their dissemination in the environment and has triggered antimicrobial resistance, which is actually a major health issue [7]. Aquaculture constitutes an important source of the antibiotics’ presence in the environment. It has been estimated that between 70% and 80% of the antibiotics administered as pelleted medicated feed in aquacultures are released into the aquatic environment due to uneaten remains or as metabolic waste products via excretion [8,9,10,11,12,13]. In the case of SDZ, it is metabolized into *N*(4)-acetyl-sulfadiazine, and both the active form and the metabolite are excreted. Then, the metabolite can itself be converted again into the active form of SDZ by hydrolysis [14]. OXA is known to be metabolized in a small extent and it is therefore frequently present in recirculating aquaculture systems (RAS) and/or is released into surface waters [15].

Photodegradation is a natural process able to reduce antibiotics’ concentration in the aquatic environment. However, its application in water treatment requires the increase of photodegradation rates and, for this purpose, the use of photocatalysts has been proposed [16,17,18,19]. Recently, carbonaceous materials have gained popularity in the design and synthesis of novel photocatalysts [20,21,22,23,24,25] due to their advantageous properties (e.g., good electron conductivity, physicochemical stability, relatively large surface area, and facile synthesis). Among them, carbon dots (CD), discovered by chance in 2004, have gradually become a rising star in the nanocarbon family and have inspired extensive studies. Compared to traditional semiconductor quantum dots, CD have the advantages of low cost, low toxicity, facile functionalization, good biocompatibility, tunable fluorescence emissions, high resistance to photobleaching, and chemical inertness, being promising alternatives in many uses [26]. The photocatalytic applications of CD include their use as pure CD or as blended materials, which aim to enhance CD’s photocatalytic activity by strategies such as doping, surface modulation, and construction of metal/CD or CD/semiconductor composites [26]. The basis of these applications is the capacity of CD and their nanocomposites to produce reactive oxygen species (ROS) and to therefore increase the photodegradation rate. Recently, such a capacity has been explored for the removal of pollutants from water [26,27,28,29,30], but this is still a field of wide opportunities for research.

Several methodologies have been proposed for the synthesis of CD, which may be divided into “top-down” and “bottom-up” methods, the latter being simpler and more economic [31]. Among the “bottom-up” methods, microwave radiation [32], chemical or thermal oxidation [33], and/or alkali-assisted ultrasonic synthesis [29], using carbon containing molecules (such as glucose, fructose, ascorbic acid) as carbon-based precursors for the preparation of CD, can be highlighted. As for hybrids, a great effort has recently been made on the preparation of novel materials comprised of CD and inorganic nanoparticle cores. For example, the nanohybrid-based photocatalysts TiO_2_/CD and SiO_2_/CD, were prepared by Hazarika and Karak (2016) [28] and Zhang et al. (2014) [34], respectively; ZnO/CD hybrid nanostructures were hydrothermally synthesized by Bozetine et al. (2016) [32]; and FeO_3_/CD nanocomposites were prepared by a facile solvent-thermal process in aqueous solution by Yu and Kwak (2012) [35]. Among the inorganic nanoparticle cores, TiO_2_ is one of the most promising options due to its high chemical stability, low cost, environmental friendliness, and optical properties [36]. Nevertheless, pure TiO_2_ presents a rapid recombination of photoexcited electron–hole pairs and is exclusively activated by UV light, which restrains its generalized application under solar radiation [30]. Therefore, the combination of CD with TiO_2_ is advantageous since allows for an enhanced light-absorption efficiency and photocatalytic performance by broadening the photo-absorption region and decreasing the recombination of electron–hole pairs [30].

In this work, CD were synthesized and coupled with TiO_2_, aiming at the evaluation of their efficiency to increase the photodegradation rate of two antibiotics widely used in aquaculture—OXA and SDZ. The applicability of the produced nanocomposites was not only evaluated in ultrapure water (UW), but also in water containing synthetic sea salts (SSS, 30‰), to simulate their behaviour in a marine aquaculture environment.

## 2. Experimental Procedure

### 2.1. Standards and Solutions

SDZ (>99%) and OXA (98%) were obtained from TCI Europe and Fisher Scientific, respectively. The SDZ standard solution (10 mg L^−1^) was prepared using 0.001 mol L^−1^ phosphate buffer at pH 8.3, while the OXA stock solution (100 mg L^−1^) was prepared by dissolving the compound in 0.5 mol L^−1^ sodium hydroxide solution and sonicated for 60 min. Then, the working solution (10 mg L^−1^) was prepared in 0.001 mol L^−1^ phosphate buffer and the pH was adjusted to 7.3. The phosphate buffer stock solution (1 L) was prepared using 0.05 mol of sodium dihydrogen phosphate dihydrate (Fluka, Biochemika, ≥99.5%) and 0.05 mol di-sodium hydrogen phosphate dihydrate (Fluka, Biochemika, ≥99%), which was then diluted to 0.001 mol L^−1^ and the pH was adjusted using hydrochloric acid (NormaPur, 37%). The SDZ and OXA standard solutions (10 mg L^−1^) were also prepared using SSS solution (30‰), obtained from Red Sea Salt (Red Sea Europe), resulting in a natural pH of 8.6.

For high-performance liquid chromatography with a UV-visible detector (HPLC-UV) analysis, methanol (Fisher Scientific, HPLC grade) and formic acid (Sigma-Aldrich, >98%) were used. UW was obtained using a purification water system (Elga Purelab Flex 4 from Elga (Veolia)).

### 2.2. Preparation of Photocatalysts

CD preparation was performed according to the one-step hydrothermal method described by Hazarika and Karak (2016) [28], with minor modifications. Succinctly, 2.5 g of citric acid (Pronolab, ≥99.5%) and 2.5 g of glycerol (Fisher Scientific, ≥99%) were dissolved in 15 mL of UW. Then, 6.6 mL of NH_4_OH (Fluka, 25% (*w*/*w*)) was added to the previous mixture, followed by 23.4 mL of UW. The solution was transferred to a Teflon-lined stainless-steel autoclave and kept at 150 °C for 4 h. A dark brown solution containing the CD was obtained, which was left to cool at room temperature. Water was then evaporated at 60 °C for 24 h in a furnace, obtaining a gelatinous material.

To obtain CD-TiO_2_ composites, two different approaches were used, ex-situ (CD/TiO_2_) and in-situ (CD@TiO_2_). In the ex-situ technique, 0.25 g of previously synthesized CD was mixed with 12.8 mL of UW, 2.2 mL of NH_4_OH and 15 mL of concentrated HCl (Fluka, 37% (*w*/*w*)) under continuous stirring for 5 min. Then, 0.8 mL of titanium(IV) *n*-butoxide (>99%, Alfa Aesar) was added dropwise and agitated vigorously for 10 min. The solution was transferred to a Teflon-lined stainless-steel autoclave and kept at 150 °C for 8 h. The autoclaves were cooled at room temperature and the solution was brought to dryness at 50 °C. In the in-situ procedure, 0.668 g of citric acid, and the same mass of glycerol, were mixed with 12.8 mL of water, 2.2 mL of NH_4_OH, and 15 mL of concentrated HCl and agitated for 10 min. Afterwards, 1.6 mL of titanium butoxide was added dropwise and the mixture was agitated vigorously for 10 min. The solution was transferred to a Teflon-lined stainless-steel autoclave and kept at 150 °C for 8 h. The autoclaves were cooled at room temperature and the solution was filtered and the precipitate was dried overnight at 50 °C.

Bare TiO_2_ was obtained by a simple hydrothermal process, by mixing 15 mL of concentrated HCl with 12.8 mL of UW and 2.2 mL of NH_4_OH, which was then stirred for 10 min. Titanium butoxide was added dropwise (0.8 mL) and the mixture was agitated vigorously for 10 min. Like before, the solution was transferred to a Teflon-lined stainless-steel autoclave and kept at 150 °C for 8 h. The autoclave was cooled at room temperature and the solution transferred to 50-mL centrifuge tubes and centrifuged at 5000 rpm for 15 min and dried at 50 °C overnight. The obtained TiO_2_ was calcinated in a muffle furnace at 300 °C for 2 h.

### 2.3. Characterization of Photocatalysts

The UV-visible spectra of aqueous solutions (100 mg L^−1^ for CD and 200 mg L^−1^ for CD@TiO_2_, CD/TiO_2_ and TiO_2_) were obtained using a T90+ Spectrometer from PG Instruments Limited, while fluorescence emission spectra were recorded using a Cary Eclipse Fluorescence Spectrophotometer from Agilent Technologies.

X-ray diffraction (XRD), conducted to evaluate the structural properties of the synthesized powders, was acquired using a Malvern Panalytical Empyrean diffractometer with the Cu (Kα) radiation in a 2θ range of 8–70°.

Fourier-transformation infrared spectroscopy-attenuated total reflectance (FTIR-ATR) was performed to analyze the photocatalysts’ surface. Each one was placed onto the diamond ATR window of an Avatar 360 Thermo Nicolet spectrometer and scanned over the range of 500–4000 cm^−1^ with a resolution of 4 cm^−1^ in absorbance mode and expressed as an average of 64 readings.

Scanning electron microscopy (SEM) images were obtained using a scanning electron microscope, analytical and high resolution Schottky emission (HR-SEM-SE), Hitachi model SU-70, equipped with energy-dispersive X-ray microanalysis (EDS), Bruker model Quantax 400. The samples were carbon-coated prior to the microscopy analysis.

### 2.4. Photodegradation Experiments

SDZ and OXA photodegradation experiments were performed in the presence and absence of the four synthesized photocatalysts (CD, CD@TiO_2_, CD/TiO_2_ and TiO_2_) at different concentrations and under simulated solar irradiation using a Solarbox 1500 (Co.fo.me.gra, Italy). The device, which contains an arc xenon lamp (1500 W) and outdoor UV filters, which limit the transmission of light to wavelengths below 290 nm, was kept at a constant irradiance of 55 W m^−2^ (290–400 nm) throughout all the experiments. Likewise, the device was refrigerated by an air-cooled system to keep a constant temperature inside. To monitor the device irradiance level and temperature, a multimeter (Co.fo.me.gra, Milano, Italy) equipped with a UV 290–400 nm large band sensor and a black standard temperature sensor, was used. Furthermore, a parabolic reflection system guaranteed the uniformity of the irradiation inside the chamber.

The antibiotics’ solutions (40 mL) were transferred into quartz tubes (1.8 cm internal diameter × 20 cm height) which were placed inside the irradiation chamber of the Solarbox using a home-made metallic holder, in order to ensure homogeneous irradiation. For each set of experiments, four tubes were placed on the holder: three of them were exposed to radiation and one was covered with several layers of aluminum foil to protect it from light (dark control). The dark control was kept inside the Solarbox for the same amount of time as the irradiated solutions. It was used to check if the concentration of antibiotics remained the same or, otherwise, to determine the occurrence of adsorption onto the nanocomposite under study and/or degradation other than that which was photo-induced (e.g., by microbiological or thermal means).

For CD, a 40 g L^−1^ stock solution was prepared. For each quartz tube, an aliquot with the appropriate volume was added to the solution to obtain the necessary photocatalyst concentration. In what concerns the other photocatalysts, the corresponding mass was weighted and added to each tube to obtain the appropriate photocatalyst concentration. Initially, SDZ solutions (10 mg L^−1^) were irradiated for 4 h in the absence and presence of a photocatalyst at concentrations ranging from 25 to 1000 mg L^−1^. For OXA solutions (10 mg L^−1^) in the absence and presence of a photocatalyst at concentrations ranging from 5 to 250 mg L^−1^, 1 h of irradiation was used. After selecting the most efficient photocatalyst as well as the best concentration of it for each antibiotic, SDZ and OXA photodegradation kinetic experiments were performed in phosphate buffer (0.001 mol L^−1^) and SSS (30‰) solutions.

For each irradiation time, aliquots from the three irradiated tubes and from the dark control were collected and analyzed by HPLC-UV. The remaining concentration of SDZ and OXA in irradiated solutions (*C*) was compared with the respective dark control (*C*_0_) for determining the percentage of degradation at each irradiation time (*t*, h), according to Equation (1):

Photodegradation (%) = *C*/*C*_0_ ∗ 100 (1)

GraphPad Prism 5 was used to determine the fittings of experimental data to the pseudo first-order kinetic equation *C*⁄*C*_0_ = e^−*kt*^, where *k* is the pseudo first-order degradation rate constant (h^−1^). Additionally, the half-life times (*t*_1/2_) of SDZ and OXA were calculated as ln2/*k*.

### 2.5. Chromatographic Analysis

A Water Alliance 2695 Separation Module equipped with a Waters 2487 Dual Absorbance detector was used for the determination of SDZ and OXA in the aqueous samples. An ACE^®^ C18 column-PFP (150 mm × 4.6 mm i.d. with 5 µm particle size) connected to a 4.6 mm i.d. ACE^®^ 5 C18 guard was used, at 25 °C, and 20 μL of each sample was injected. Three replicate injections were carried out for each determination, under a mobile phase flow rate of 0.9 mL min^−1^. The mobile phase consisted of a mixture of methanol: 0.1% formic acid, 20:80 (*v*/*v*) or 45:55 (*v*/*v*) for the analysis of SDZ and OXA, respectively, and both compounds were detected at 270 nm. Before use, methanol and 0.1% formic acid aqueous solutions were filtered through a 0.2 μm polyamide membrane filter (Whatman).

## 3. Results and Discussion

### 3.1. Characterization of Photocatalysts

#### 3.1.1. CD Characterization

**Figure 1:**
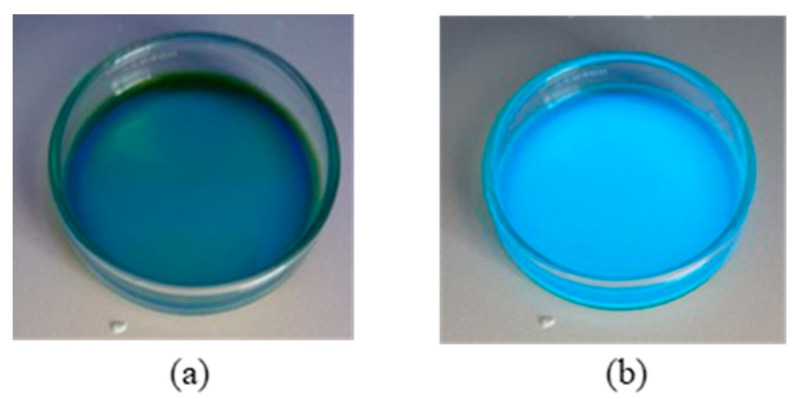
CD under UV light at (**a**) 254 nm and (**b**) 365 nm.

**Figure 2:**
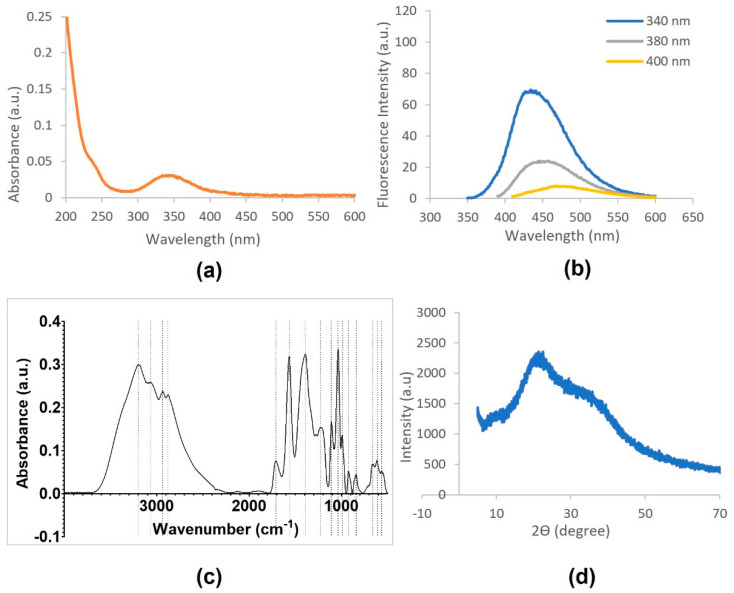
Characterization of CD by (**a**) UV-visible absorption; (**b**) fluorescence; (**c**) FTIR-ATR; and (**d**) XRD.

The one-step synthesis of CD was performed by carbonization of citric acid and glycerol in the presence of a source of nitrogen obtained from NH_4_OH. As mentioned by Hazarika and Karak (2016) [28], the use of a nitrogen source introduces fluorescence characteristics without the need of any additional passivating agent. Under UV light at 254 nm, the CD presented some fluorescence (Figure 1a), however at 365 nm an intense blue light was emitted (Figure 1b), confirming the results obtained by Hazarika and Karak (2016) [28]. Such contrasting fluorescence may be related to the different absorbance at these two wavelengths, as displayed by the UV-visible spectrum of CD (Figure 2a).

In Figure 2a, the UV-visible absorption spectrum of CD (100 mg L^−1^ in water) demonstrates a high absorbance for wavelengths below 400 nm. Particularly, a significant peak at 350 nm is observed, characteristic of n-π* transition of C=O, along with a shoulder at 230 nm attributed to π-π* transitions of the nitrogen heterocyclic *sp*^2^ moieties [28,33,37,38].

Fluorescence spectra of the CD are presented in Figure 2b, where it is possible to verify an emission band at around 350–600 nm, with excitation wavelengths of 340, 389, and 400 nm, demonstrating the wavelength-dependent nature of CD emission. The maximum intensity of emission was observed at 425 nm, using an excitation wavelength of 340 nm. Moreover, with the increase from 340 to 400 nm, fluorescence intensity decreased accordingly and shifted to longer wavelengths. The difference in the position of the emission peak might be due to CD size variation, while the intensity depends on the number of particles that were excited at that particular wavelength. On the other hand, surface defects were also considered responsible for controlling the fluorescence mechanism [28].

The FTIR-ATR spectra of CD, presented on Figure 2c, comprise a broad absorption band centered at 3200 cm^−1^, which can be attributed to O–H stretching [33], implying that CD present a large number of residual hydroxyl groups on its surface [28]. The absorption band at 2936 cm^−1^ and 2880 cm^−1^ can be attributed to C–H symmetric stretching, while the band at 1710 cm^−1^ can be assigned to stretching vibrations of the C=O group [28]. Moreover, CD FTIR-ATR spectra reveal characteristic peaks of –NH (1562 cm^−1^), and C–NH–C (1110 cm^−1^) [38]. Peaks generally attributed to C–C vibrations of oxygen-containing groups can be found (1390 and 1038 cm^−1^), and the band at 924 cm^−1^ may be due to the epoxy group [28,32]. Finally, the peak near 1200 cm^−1^ can be attributed to the alkoxy C–O–C stretching [33]. The presence of the polar functional groups containing either oxygen or nitrogen in the surface explain the highly hydrophilic characteristics and the good solubility of the CD in water.

The XRD pattern of CD (Figure 2d) showed a broad single peak around 21° (2Ɵ), similarly to the CD synthesized by Hazarika and Karak (2016) [28] or by other approaches [27,32]. This broad peak is related to highly disordered carbon atoms and a predominantly amorphous nature.

#### 3.1.2. CD Nanocomposites Characterization

**Figure 3:**
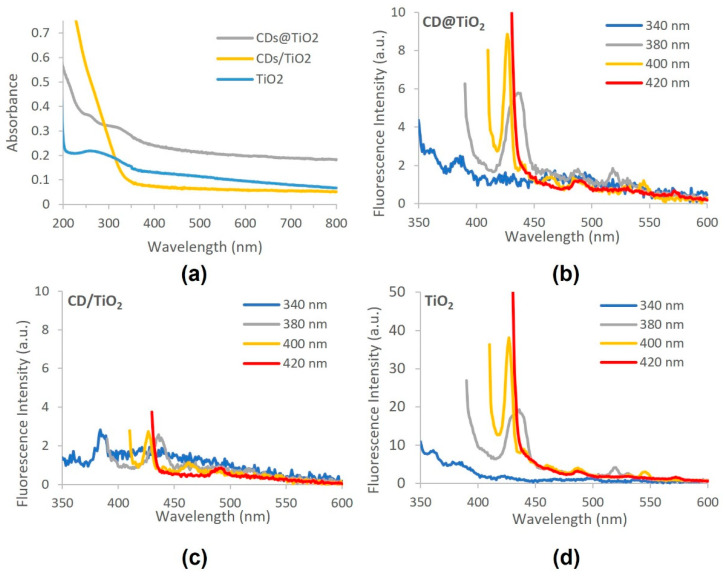
CD nanocomposites characterization by (**a**) UV-visible absorption (CD@TiO_2_, CD/TiO_2_ and TiO_2_); and (**b**–**d**) fluorescence (CD@TiO_2_ (**b**); CD/TiO_2_ (**c**); TiO_2_ (**d**)).

Figure 3a presents the UV-visible absorption spectra of CD@TiO_2_, CD/TiO_2_ and TiO_2_ (200 mg L^−1^ in water). TiO_2_ nanoparticles presented a broad absorption band from 200 to 350 nm, demonstrating its ability to absorb radiation, which is then used in the photodegradation process. The incorporation of CD in the TiO_2_ by the in-situ approach (CD@TiO_2_) originates a slight shift of the absorption to higher wavelengths and increases the absorption of the radiation when compared to absorption observed for TiO_2_. On the other hand, CD/TiO_2_ presented higher absorbance than the other materials at wavelengths lower than 350 nm, but less in the region of the solar spectrum, which may be related to the ex-situ procedure used to synthesize this composite.

**Figure 4:**
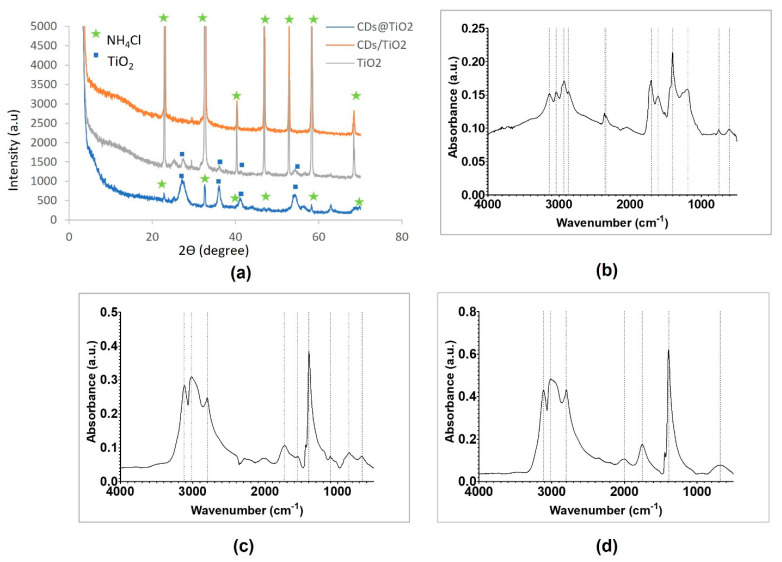
CD nanocomposites characterization by (**a**) XRD (CD@TiO_2_, CD/TiO_2_, and TiO_2_); and by (**b**–**d**) FTIR-ATR (CD@TiO_2_ (**b**), CD/TiO_2_ (**c**), and TiO_2_ (**d**)).

The maximum emission peaks in the fluorescence spectra of CD@TiO_2_, CD/TiO_2_, and TiO_2_ were observed at 427 nm at an excitation of 400 nm (Figure 4b–d). A higher fluorescence intensity was observed for TiO_2_ alone, followed by CD@TiO_2_ and CD/TiO_2_. The lower intensity observed for CD/TiO_2_ might be related to the lower absorbance observed at 400 nm (Figure 4a). As previously observed for CD (Figure 2b), for all the materials in Figure 3b–d, fluorescence emission depends on the excitation wavelength, probably due to the existence of different surface states and size dispersions of the nanomaterials. Notably, no emission attributed to CD nanoparticles was observed for the CD/TiO_2_, nor for the CD@TiO_2_ material, as it was observed for the CD alone (Figure 2b), with an emission maximum observed at 425 nm when using an excitation wavelength of 340 nm. The emission spectra of the CD/TiO_2_ and CD@TiO_2_ materials is similar in shape to the one observed for TiO_2_, which seems to suggest that TiO_2_ particles are the only ones contributing to the emission process.

The CD@TiO_2_, CD/TiO_2_, and TiO_2_ XRD pattern (Figure 4a) showed that, for all materials, NH_4_Cl was present in the final photocatalyst identified by the International Centre for Diffraction Data (ICDD) database, with the reference code 01-075-6255 and the peaks around 22.8°, 32.6°, 40.5°, 46.8°, 58.1°, and 68.5°. Moreover, the CD@TiO_2_ and TiO_2_ XRD pattern also showed the presence of TiO_2_, identified by the ICDD database with the reference code 04-006-1930 and with peaks around 27.3°, 36.1°, 41.2°, and 54.2° assigned to the (110), (101), (111), and (211) planes of rutile TiO_2_, respectively [39,40]. On the other hand, CD/TiO_2_ did not present any other peak that could identify TiO_2_, which could be due to the high percentage of NH_4_Cl relative to TiO_2_ revealed in the EDS results (Table 1) (this way, the crystalline phase of NH_4_Cl may disguise the crystalline phase of TiO_2_ present in the material).

For CD@TiO_2_ (Figure 4b), CD/TiO_2_ (Figure 4c), and TiO_2_ (Figure 4d), it is possible to identify some equal or similar absorption peaks.

An absorption band with values among 3136 and 3110 cm^−1^, as explained for CD, can be attributed to O–H stretching from water molecules adsorbed on the particles’ surface [41]. The values 3040 and 3020 cm^−1^ can correspond to the stretch of the C–H bond of alkenes, while 2800 cm^−1^ is probably due to the C–H bond stretching from an aldehyde [33]. The absorption bands at 1754 and 1734 cm^−1^ can be assigned to stretching vibrations of the C=O group; meanwhile, the stretching of CH_3_ is usually associated with peaks between 1400 and 1388 cm^−1^ [28,32]. In CD@TiO_2_ and CD/TiO_2_, the absorption peaks at 1190 and 1100 cm^−1^, respectively, are associated with C–O stretching of ester, and the absorption bands at approximately 750, 680, and 660 cm^−1^ in CD@TiO_2_, TiO_2_, and CD/TiO_2_, respectively, correspond to the stretching vibration of the Ti–O–Ti group [41,42].

**Figure 5:**
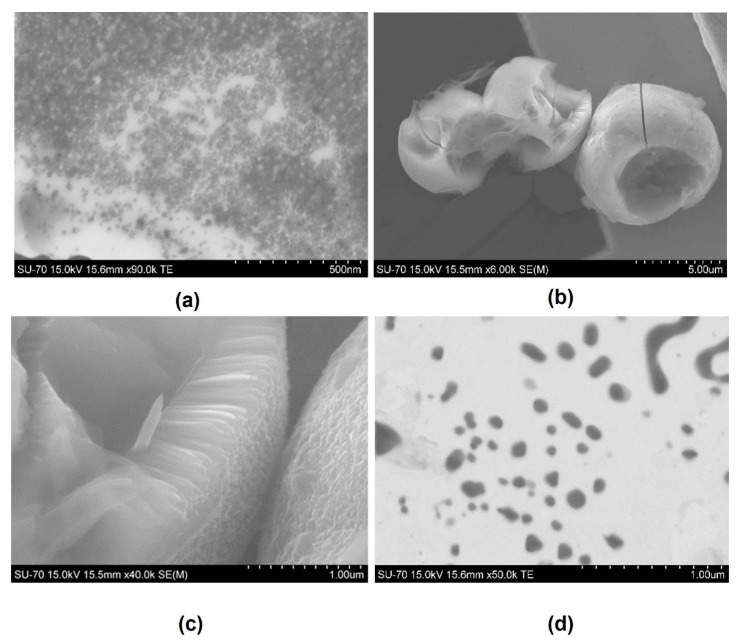
SEM images for (**a**) TiO_2_, (**b**,**c**) CD@TiO_2_, and (**d**) CD/TiO_2_.

For CD@TiO_2_ in particular, shown in Figure 4b, it was possible to identify more absorption bands. This could be attributed to the synthesis method of this photocatalyst. In this case, absorption band around 2940 cm^−1^ is associated with the symmetric stretching of C–H groups, while 2870 cm^−1^ is associated with C–H bond of primaries or secondaries CH_3_ and CH_2_ [28]. In this material, the peaks around 2350 cm^−1^ indicated the C=O group, identified as CO_2_ from the room atmosphere during the spectra acquisition. As for CD, 1700 cm^−1^ can correspond to stretching vibrations of the C=O group [28], while 1610 cm^−1^ is attributed to the bending vibration of the Ti–O bond in TiO_2_ [41,42]. Finally, the absorption band of 606 cm^−1^ may be associated with C–Cl bond stretching provided by the hydrochloric acid used during the synthesis. On the other hand, CD/TiO_2_ (Figure 4c) presented two absorption bands that could be associated with N–O bond stretching of NO_2_ since they are strong asymmetric and symmetric axial deformation bands (1550 and 1398 cm^−1^). For TiO_2_ (Figure 4d), another absorption band at 2000 cm^−1^ was found, which is normally associated with the C=C=N group stretching. The occurrence of groups with carbon in TiO_2_ suggested the presence of the original compound used as a TiO_2_ precursor, namely, titanium(IV) n-butoxide. Furthermore, the new groups in CD@TiO_2_ and CD/TiO_2_ indicated that TiO_2_ was modified and functionalized by CD during the adopted synthesis methodologies. The preparation of bare TiO_2_ originated small TiO_2_ nanoparticles typically smaller than 20 nm (Figure 5a).

The synthesis of the TiO_2_ particles, being in-situ or ex-situ, has a great influence on the morphology of the final structures obtained. While the TiO_2_ particles in CD@TiO_2_ are observed in the form of thin leaf nanoparticles organized into spherulites (Figure 5b,c), in CD/TiO_2_ they are present in the form of small particles dispersed in the carbon film of the TEM grid (Figure 5d). These particles are, however, bigger than the ones observed for the bare TiO_2_ nanoparticles, and show sizes that can range from 30 to more than 100 nm. The presence of already-formed CD nanoparticles, in the case of the synthesis of ex-situ CD/TiO_2_, clearly hampers particle growth, and only small sized (typically submicrometric) particles were obtained. The concurrent synthesis promotes TiO_2_ particle growth by the formation of larger individual particles, but also seems to promote the controlled aggregation, as observed in the case of the CD@TiO2 (Figure 5d).

The EDS results obtained for the different materials are presented in Table 1 and confirm the formation of TiO_2_, although the extension of such a synthesis appears to be different. A high content of chlorine, probably due to the HCl used in the synthesis, can be observed for CD@TiO_2_ and CD/TiO_2_ and might result from the presence of NH_4_Cl as secondary product of the TiO_2_ formation. These results are in agreement with the presence of NH_4_Cl observed in the XRD pattern (Figure 4a).

### 3.2. Photodegradation Experiments

#### 3.2.1. Evaluation of the Photocatalyst Performance

**Figure 6:**
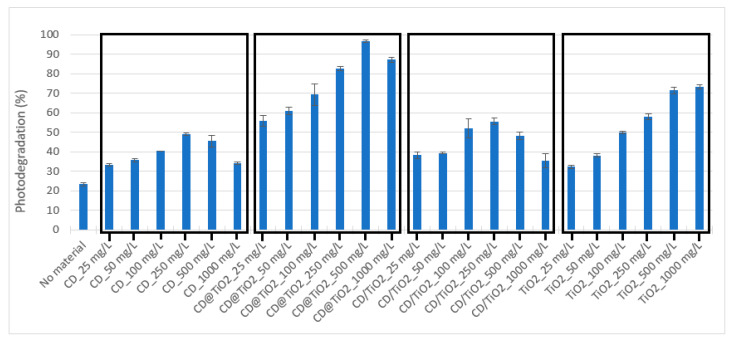
SDZ (10 mg L^−1^) photodegradation (%) after 4 h of simulated solar radiation in the absence and presence of photocatalysts at concentrations ranging from 25 to 1000 mg L^−1^. Note: Error bars represent standard deviations (*n* = 3).

In order to verify the photocatalytic efficiency of each synthesized material, SDZ solutions (10 mg L^−1^) were irradiated for 4 h in absence of the photocatalyst, and in its presence, at concentrations ranging from 25 to 1000 mg L^−1^. SDZ concentration remained stable in dark controls, which demonstrated that no adsorption of the SDZ onto the nanocomposites, or degradation other than that which was photo-induced, occurred. The results obtained can be observed in Figure 6, and show that for all the catalysts tested, higher photodegradation percentages were reached in comparison to the ones obtained in the absence of photocatalysts. Moreover, for the materials containing CD, photodegradation increased, with photocatalyst concentration reaching a maximum before decreasing.

This trend may be related to the inner filter effect observed for carbon materials, which overlaps their photosensitizing capacity, so the net effect of CD may be dependent on their concentration. In the case of TiO_2_, photodegradation increased with its concentration until 500 mg L^−1^, from which point no further increase was observed. Yadav et al. (2018) [43] also observed that the increase in TiO_2_ concentration beyond a certain extent did not result in further improvement in SDZ removal, which was related to the turbidity increase and to the UV radiation scattering and screening, due to the catalyst particles’ agglomeration at larger concentrations. Among the four tested materials, larger photodegradation was obtained using CD@TiO_2_ at 500 mg L^−1^, with photodegradation increasing from 23 ± 1% in the absence of a photocatalyst to 97 ± 1% in the presence of a photocatalyst.

**Figure 7:**
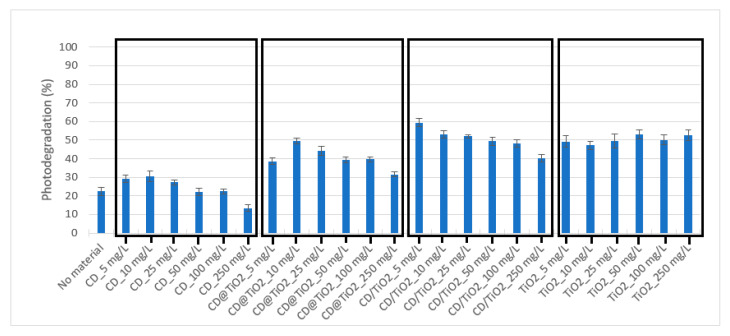
OXA (10 mg L^−1^) photodegradation (%) after 1 h of simulated solar radiation in the absence and presence of photocatalysts at concentrations ranging from 5 to 250 mg L^−1^. Note: Error bars represent standard deviations (*n* = 3).

Similar to the experiments performed for SDZ, OXA solutions (10 mg L^−1^) were irradiated for 1 h in the absence and presence of photocatalysts at concentrations ranging from 5 to 250 mg L^−1^. Again, as for SDZ, OXA concentration remained stable in dark controls, demonstrating that no adsorption onto the nanocomposites or degradation other than that which was photo-induced occurred during the experiments performed. The obtained results are depicted in Figure 7. In this case, the increase in photodegradation associated with the presence of CD materials was less pronounced than for SDZ. Furthermore, a decreasing trend was observed with the increase of CD nanocomposite concentrations, which may be related to the higher inner filter effect.

Concerning TiO_2_, the results were similar for all the concentrations tested (Figure 7). The material with better photocatalytic results was CD/TiO_2_, which provided OXA photodegradation percentages between 40 and 59%. Louros et al. (2020) [44] reported a decrease in the OXA photodegradation rate in the presence of organic matter due to its inhibitory effect by acting as a filter (inner filter effect), decreasing the radiation available for the pollutant. In the case of CD, the higher absorbance observed in the UV-visible spectrum of CD@TiO_2_ (Figure 3a) may be related to a larger inner filter effect and with the lower photodegradation percentages attained for OXA, in comparison with CD/TiO_2_ and TiO_2_, as well as the lower efficiency of CD composites observed at higher concentrations. Therefore, for OXA, the inner filter effect of CD materials may have surpassed their photosensitizing effect at lower concentrations more than for SDZ. From all the four materials tested, the most remarkable photocatalytic results for OXA were obtained using CD/TiO_2_ at 5 mg L^−1^, with an increase from 22 ± 2% to 59 ± 2%, in the absence and presence of a photocatalyst, respectively.

The above results evidenced that CD and TiO_2_ composites exhibited a more remarkable photocatalytic performance than pristine TiO_2_ for either SDZ or OXA. To the best of our knowledge, there are no published results on the application of such composites to the solar-driven photocatalytic removal of SDZ or OXA. However, the improved capacity of CD and TiO_2_ composites as compared with TiO_2_ has already been observed for other antibiotics, such as ciprofloxacin [30] or levofloxacin [45], where a relation between the enhanced efficiency of photocatalysts, with the extra active sites provided by CD, and the restriction of the recombination of charge carriers was proposed. In any case, this is the first work to compare the photocatalytic efficiency of CD and TiO_2_ composites prepared under different procedures in the degradation of antibiotics. In this way, it was possible to show that the efficiency of the different tested photocatalysts, including CD@TiO_2_ and CD/TiO_2_, was different for SDZ and OXA. This may be justified by the multiple specific processes involved in the photocatalytic degradation of any organic compound, which comprise adsorption–desorption, electron–hole pair production, the recombination of electron pairs, and chemical reactions.

#### 3.2.2. Evaluation of the Photocatalyst Performance

**Figure 8:**
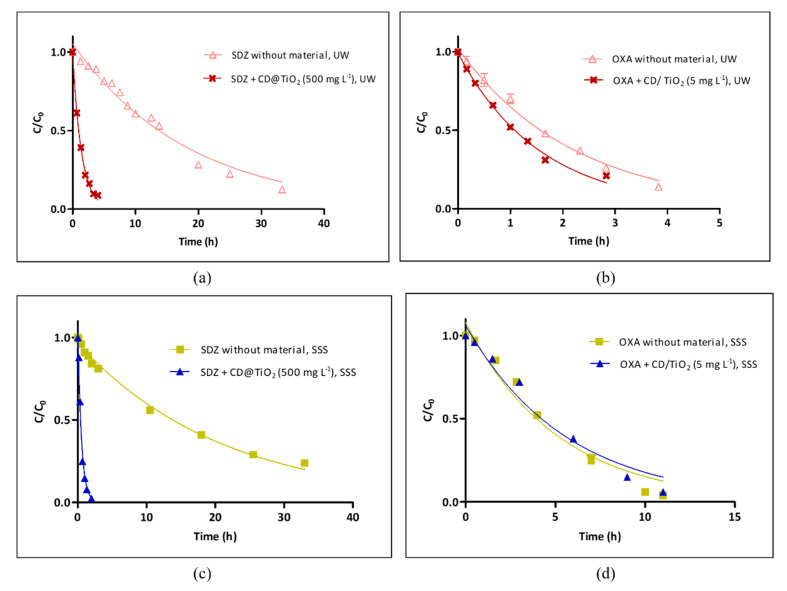
Experimental results on the kinetics of antibiotics photodegradation (*C*i = 10 mg L^−1^) in the presence and absence of the photocatalyst, together with the curves of pseudo first-order decay fitted to the data by nonlinear regression obtained for (**a**) SDZ in UW; (**b**) OXA in UW; (**c**) SDZ in SSS; and (**d**) OXA in SSS (Note that, for most of the experimental points, error bars are too small to be visible).

Kinetic experiments performed for SDZ and OXA in UW and in SSS, in the absence and presence of the most efficient photocatalyst at the concentration identified in the previous section, are presented in Figure 8 together with the curves of pseudo first-order decay, fitted to the data by nonlinear regression. The corresponding parameters of *k* (h^−1^), the determination coefficient (*r*^2^), and the *t*_1/2_ (h) are presented in Table 2.

In agreement with results observed in the previous section, the photocatalytic performance of the CD and TiO_2_ composites was more notable for SDZ (Figure 8a) than for OXA (Figure 8b). The *k* obtained for SDZ in UW increased from 0.054 ± 0.003 h^−1^ in the absence of a photocatalyst to 0.71 ± 0.02 h^−1^ in the presence of 500 mg L^−1^ of CD@TiO_2_, which resulted in a decrease in the correspondent *t*_1/2_ from 12.8 ± 0.8 h to 0.98 ± 0.03 h (Table 2). For OXA, although the effect of the presence of a photocatalyst was not so pronounced, the *k* in UW increased from 0.045 ± 0.03 h^−1^ in the absence of a photocatalyst to 0.63 ± 0.03 h^−1^ in the presence of 5 mg L^−1^ of CD/TiO_2_, which resulted in a decrease in the correspondent *t*_1/2_ from 1.54 ± 0.07 h to 1.10 ± 0.05 h (Table 2). The *t*_1/2_ are strictly related to experimental conditions, but, assuming that the lamp properly simulates sunlight, results can be converted into outdoor *t*_1/2_, in sunny summer days (SSD) equivalents (Table 2). Considering that the total energy reaching the ground on a SSD (45° N latitude) is 7.5 × 10^5^ J m^−2^, one SSD (24 h day/night cycle) will correspond to 3.8 h of irradiation with the present Solarbox equipment [46]. This conversion allows us to determine the *t*_1/2_ values at environmentally relevant conditions. For SDZ, the application of the photocatalyst in UW resulted in a decrease of 3.4 ± 0.2 SSD to 0.258 ± 0.008 SSD, while for OXA, that reduction was only from 0.41 ± 0.02 SSD to 0.29 ± 0.01 SSD (Table 2). Therefore, for both antibiotics, the obtained *t*_1/2_ values using the selected photocatalysts were lower than 0.30 SSD.

In order to ascertain the application of the produced photocatalysts in aquaculture systems containing salt water, the same conditions that were applied in UW were tested for SDZ and OXA photodegradation in SSS water solutions. The results obtained for each of the considered antibiotics were quite different (Figure 8c,d). In the absence of a photocatalyst, the *k* obtained for SDZ decreased from 0.054 ± 0.003 h^−1^ in UW to 0.048 ± 0.002 h^−1^ in SSS (Table 2). However, the photocatalyst performance improved in SSS, with *k* increasing from 0.71 ± 0.02 h^−1^ in UW to 1.8 ± 0.2 h^−1^ in SSS. This could be associated with the SDZ speciation with pH (in SSS, at pH 8.6, SDZ is anionic, while in UW, at 7.3, both uncharged and anionic species coexist). In fact, the increase of the SDZ photodegradation rate with pH due to the presence of its anionic form has already been observed [47]. As for the *t*_1/2_, 500 mg L^−1^ of CD@TiO_2_ in SSS provided a decrease from 14.5 ± 0.7 h (in absence of material) to 0.38 ± 0.04 h. For OXA, similarly to SDZ, *k* decreased from 0.45 ± 0.02 h^−1^ in UW to 0.20 ± 0.02 h^−1^ in SSS in the absence of a catalyst. However, in this case, the photocatalyst performance was nullified in SSS, so the *t*_1/2_ remained the same in the absence (3.5 ± 0.3 h) and presence of CD/TiO_2_ (3.9 ± 0.4 h). This may be related to the photocatalyst concentration, which was used in SSS for comparison purposes but had been optimized for UW. It is also known that ionic substances, such as Cl^−^, CO_3_^2−^, HCO_3_^−^, NO_3_^−^, NO_2_^−^, and PO_4_^3−^, may affect photoelectron generation, electron–hole recombination, and ^•^OH radical scavenging [48], which may be underneath the observed results. Furthermore, in the specific case of TiO_2_ photocatalytic activity, apart from radical and hole scavenging, fouling effects of inorganic ions have been related to UV screening, competitive adsorption to surface active sites, competition for photons, surface deposition of precipitates and elemental metals, and a direct reaction with the photocatalyst [49]. For example, Sirtori et al. (2010) [50] observed that the presence of ions in seawater inhibited the photocatalytic degradation of trimethoprim. This effect was mostly related to the scavenging of the generated ^•^OH radicals by Cl^-^ ions in seawater [50], which may have also affected OXA photodegradation in SSS under the presence of CD/TiO_2_.

This work is a novel contribution on the application of CD and TiO_2_ composites to the solar-driven photocatalytic removal of aquaculture antibiotics from water. The synthesized composites were here shown to be more efficient than TiO_2_ in the degradation of SDZ and OXA, but differences in efficiency were observed for these antibiotics; for CD@TiO_2_ (in-situ procedure) and CD/TiO_2_ (ex-situ procedure); and for the two considered matrices, namely, UW and SSS. Considering the promising obtained results, and in order to shed light on the observed differences, future work on the characterization of the synthesized materials should be carried out (e.g., X-ray photoelectron spectroscopy (XPS) to identify surface chemical states, nitrogen adsorption–desorption isotherms to determine specific surface area and porosity, electron spin resonance (ESR) spectroscopy to figure out oxide radicals’ production). In addition, photoproducts from SDZ and OXA degradation, and the photocatalytic mechanisms under the utilization of each material, are to be determined. Finally, in view of the practical implementation of these photocatalysts, their after-use separation, stability, and reusability should be assessed. The referred-to complementary studies will be essential to prove the feasibility of using the produced composites for a sustainable and green treatment of aquaculture effluents aimed at the removal of antibiotics.

## 4. Conclusions

The main objective of this work was to investigate the photodegradation of two antibiotics applied in aquaculture—SDZ and OXA—using a solar radiation simulator in the presence of CD coupled with TiO_2_. Globally, four different materials were produced and tested for the photodegradation of SDZ and OXA: CD, CD@TiO_2_, CD/TiO_2_, and TiO_2_ alone. The materials synthesized enhanced the photodegradation of both antibiotics. CD@TiO_2_ at 500 mg L^−1^ for SDZ, and CD/TiO_2_ at 5 mg L^−1^ for OXA, were found responsible for the highest photodegradation. The kinetic results obtained using different water matrices demonstrated that the photocatalytic performance of the CD-TiO_2_ materials was more notable for SDZ than for OXA. The results obtained for OXA in SSS showed that the photocatalyst performance was annulled. However, it was in SSS that SDZ presented the lowest *t*_1/2_, demonstrating the importance of the water matrix in these studies. Concluding, photocatalysis using eco-friendly CD-TiO_2_ hybrid materials constitutes a promising and sustainable strategy to facilitate antibiotics’ efficient removal from aquaculture effluents.

## Figures and Tables

**Table 1:** EDS quantification results of the different materials.

Sample	Titanium (*at*%)	Oxygen (*at*%)	Chlorine (*at*%)	Carbon (*at*%)
**CD@TiO_2_**	13.04 ± 1.29	30.2 ± 7.80	2.14 ± 0.27	54.39 ± 8.07
**CD/TiO_2_**	2.32 ± 0.34	7.98 ± 2.21	31.56 ± 1.20	58.14 ± 9.01
**TiO_2_**	4.88 ± 0.58	10.21 ± 3.18	28.19 ± 1.45	56.72 ± 10.74

**Table 2:** Data on pseudo-first order rate constants (*k* (h^−1^)), determination coefficients (*r*^2^), half-life times (*t*_1/2_ (h)), and *t*_1/2_ converted to sunny summer days (SSD) equivalents, obtained for different matrices under simulated solar radiation (SD stands for the standard deviation, *n* = 3).

	*k* ± SD (h^−1^)	*r* ^2^	*t*_1/2_ ± SD (h)	SSD ± SD
SDZ, UW	0.054 ± 0.003	0.9769	12.8 ± 0.8	3.4 ± 0.2
SDZ + CD@TiO_2_, UW	0.71 ± 0.02	0.9973	0.98 ± 0.03	0.258 ± 0.008
SDZ, SSS	0.048 ± 0.002	0.9915	14.5 ± 0.7	3.8 ± 0.2
SDZ + CD@TiO_2_, SSS	1.8 ± 0.2	0.9803	0.38 ± 0.04	0.10 ± 0.01
OXA, UW	0.45 ± 0.02	0.9925	1.54 ± 0.07	0.41 ± 0.02
OXA + CD/TiO_2_, UW	0.63 ± 0.03	0.9937	1.10 ± 0.05	0.29 ± 0.01
OXA, SSS	0.20 ± 0.02	0.9646	3.5 ± 0.3	0.91 ± 0.09
OXA + CD/TiO_2_, SSS	0.18 ± 0.02	0.9693	3.9 ± 0.4	1.0 ± 0.1

^1^ SSD—Sunny summer days.
